# Orthographic Activation in L2 Spoken Word Recognition Depends on Proficiency: Evidence from Eye-Tracking

**DOI:** 10.3389/fpsyg.2016.01120

**Published:** 2016-07-27

**Authors:** Outi Veivo, Juhani Järvikivi, Vincent Porretta, Jukka Hyönä

**Affiliations:** ^1^Department of French, University of TurkuTurku, Finland; ^2^Department of Linguistics, University of AlbertaEdmonton, Alberta, Canada; ^3^Department of Psychology, University of TurkuTurku, Finland

**Keywords:** orthographic effects, spoken word recognition, L2, visual world paradigm, eye-tracking

## Abstract

The use of orthographic and phonological information in spoken word recognition was studied in a visual world task where L1 Finnish learners of L2 French (*n* = 64) and L1 French native speakers (*n* = 24) were asked to match spoken word forms with printed words while their eye movements were recorded. In Experiment 1, French target words were contrasted with competitors having a longer (<base> vs. <bague>) or a shorter word initial phonological overlap (<base> vs. <bain>) and an identical orthographic overlap. In Experiment 2, target words were contrasted with competitors of either longer (<mince> vs. <mite>) or shorter word initial orthographic overlap (<mince> vs. <mythe>) and of an identical phonological overlap. A general phonological effect was observed in the L2 listener group but not in the L1 control group. No general orthographic effects were observed in the L2 or L1 groups, but a significant effect of proficiency was observed for orthographic overlap over time: higher proficiency L2 listeners used also orthographic information in the matching task in a time-window from 400 to 700 ms, whereas no such effect was observed for lower proficiency listeners. These results suggest that the activation of orthographic information in L2 spoken word recognition depends on proficiency in L2.

## Introduction

A number of studies demonstrate an orthography influence on speech processing (Frost and Ziegler, 2007). As this is the case for the native language (L1) speakers who usually are exposed more to spoken than written language, we can assume that orthography could have an even more significant role in learning a second or foreign language (hereafter L2), especially in instructed learning contexts where learners are largely exposed to written language from the initial stages of the learning process. Our aim in the present study is to investigate how late L2 learners from a classroom learning background, with different proficiency levels, use orthographic information when it is explicitly available during the recognition of spoken L2 words. As the balance between orthographic and phonological knowledge is likely to be different at different proficiency levels with this type of L2 learners, we are especially interested to see whether the use of orthographic information depends on proficiency.

Orthographic activation during listening to spoken language has been observed with L1 speakers both in metaphonological tasks and in tasks that do not require an explicit analysis of the sublexical phonology. Metaphonological tasks include, for example, rhyme detection and phoneme detection. There is evidence that it is faster to detect a rhyme for word pairs with a similar spelling than for word pairs with a different spelling (Seidenberg and Tanenhaus, 1979), and that participants report hearing sounds which are not pronounced, if the corresponding letter is present in the spelling (Hallé et al., 2000). It is likely that orthographic effects obtained in this type of tasks result from different strategies of focusing on the sound structure of target words rather than from automatic activation (Damian and Bowers, 2009). However, there is also evidence for the automatic activation of orthography from tasks that do not demand an analysis of the phonological structure of spoken words. For example, the degree of orthographic consistency—whether there are multiple spellings for one sound—has been observed to influence the speed of lexical processing both in behavioral studies (Ziegler and Ferrand, 1998) and in studies measuring brain activity with event related potentials (ERPs; Perre and Ziegler, 2008; Pattamadilok et al., 2009; Peereman et al., 2009; Perre et al., 2009). Further evidence for automatic activation of orthography during spoken word processing comes from studies where orthography is present, but masked form conscious processing, like auditory lexical decision with masked visual priming (Grainger et al., 2003).

Eye-tracking provides a tool for studying the activation of phonological and orthographic information during spoken word recognition. In the visual world eye-tracking paradigm (Cooper, 1974; Tanenhaus et al., 1995; Allopenna et al., 1998; for a review, see Huettig et al., 2011) participants' eye-movements to visual cues presented on computer screen are recorded while they listen to spoken language. As the spoken words unfold, eye movements to potential referents are thought to reveal the time course of activation of different types of information during the recognition process. In the visual world paradigm, pictures are typically used as referents but also printed words can be used (cf. McQueen and Viebahn, 2007). Previous studies suggest that with printed words, language-mediated visual search is based mainly on similarities at word form level, as only phonological—but neither semantic nor visual form—competitors have been shown to yield significant competition effects (Huettig and McQueen, 2007, 2011). Printed referents can also induce semantic competition but it has been observed only in the absence of phonologically similar competitors (idem).

With L1 listeners in the visual world paradigm, in addition to testing phonological similarity between spoken words and printed referents, printed words have also been used to investigate how the degree of orthographic similarity affects the matching process (Salverda and Tanenhaus, 2010). The authors studied L1 English listeners, and observed a significant effect for the degree of orthographic similarity (bead–bear vs. bead–bare), but not for the degree of phonological similarity (bead–bean vs. bead–bear). This orthographic effect emerged only 200 ms after the onset of target words in the time-window where the body vowel was processed. Their conclusion was that orthographic information is activated immediately upon hearing spoken words, and that the matching between spoken and written English words with L1 listeners is mediated via orthography rather than phonology. ERP studies measuring brain activity have also provided further evidence for rapid of orthography in spoken word processing. For example, Perre and Ziegler (2008) observed significant changes in brain activity 200 ms after inconsistent spellings in a spoken word lexical decision task with L1 English speakers.

Orthographic effects in L1 spoken word processing have been explained to result from the acquisition order of word forms in the two modalities. According to the *offline account* (or *restructuration account*), the firstly learned phonological representations are restructured by orthographic information during literacy acquisition, and orthographic effects therefore arise within the phonological system (Taft and Hambly, 1985; Taft, 2006, 2011; Taft et al., 2008). There is evidence for this view also from brain studies where no activation has been observed in the visual processing areas during spoken language processing (Perre et al., 2009; Pattamadilok et al., 2010). In other studies using metaphonological tasks (Booth et al., 2002; Yoncheva et al., 2010), activation has been observed both in brain areas specialized in speech processing and in areas specialized in visual processing. The mechanisms of orthographic activation may therefore be also task-dependent. In contrast, the *online co-activation* account which is based on interactive-activation models of language processing (see McClelland and Rumelhart, 1981 for L1 and Dijkstra et al., 1998 for L2), claims that the processing of either written or spoken language automatically co-activates word forms also in the other modality via lexical and sublexical links (Grainger and Ferrand, 1996; Ziegler and Ferrand, 1998; Grainger et al., 2003; Ziegler et al., 2003).

The first fundamental difference between late L2 learners and L1 speakers is that L2 learners have already learned one sound system before the L2. In consequence, the perception of L2 sounds can be influenced by the L1 sound system. For example, sound contrasts absent in the L1 can be difficult to discriminate in the L2 (for a review on different types of difficulties with non-native contrasts, see Best and Tyler, 2007).

Second, as late L2 learners in instructed learning are literate, they have also learned how the sounds of the L1 are represented in spelling. These grapheme-phoneme relationships that are established during the process of learning to read in the L1 can also influence the perception of L2 sounds (Bassetti, 2006; Escudero et al., 2008; Escudero and Wanrooij, 2010; Hayes-Harb et al., 2010). Perceptual problems at the sublexical level can have consequences on the processing of spoken language at the lexical level as well. Learners may have difficulties in recognizing spoken L2 words if they have problems in recognizing individual L2 sounds.

The third important difference between early L1 acquisition and late L2 learning is that literate learners are exposed to written language in the classroom early in the learning process. Unlike for L1 speakers, who learn the written forms of words only when their spoken word forms are well established, L2 vocabulary learning is based on both written and spoken modalities. As the acquisition of orthographic information for literate L2 learners proceeds in parallel with the acquisition of phonological information or even precedes it, lexical knowledge is likely to be *co-structured* with input from the two modalities (Veivo and Järvikivi, 2013).

Orthographic activation may also depend on proficiency in L2. It is likely that in a classroom learning context where learners are exposed more frequently to written than to spoken language, beginning L2 learners have a stronger orthographic and a weaker phonological component in their lexical knowledge. As the learners are exposed more extensively to spoken language and become more proficient, the phonological component strengthens and the two modalities become better balanced. In a recent study, Veivo et al. (2015) showed that L3 learners with a formal instruction background are more accurate and more confident about word meanings in writing than in speech. Additionally, the difference between the written and spoken modalities was significantly smaller for more proficient learners. Orthographic effects in on-line tasks have also been shown to depend on proficiency; using a lexical decision task with cross-modal masked priming Veivo and Järvikivi (2013) showed that both within-language (L2) repetition primes, and between-language (L1 → L2) primes with shared orthography, produced stronger effects with higher proficiency learners. This finding suggests that there is more cross-modal activation during spoken word processing for more advanced learners and that they have a better balance between orthographic and phonological lexical knowledge in the L2 than do lower proficiency learners. This difference in the balance between modalities may also lead to qualitatively different mechanisms of orthographic activation at different proficiency levels. Beginning L2 learners may co-activate orthography in spoken word recognition sublexically, whereas more advanced L2 learners may have integrated orthographic and phonological information to common abstract representations leading to orthographic activation at the whole word level.

The results presented above show that in a task where orthography is masked from conscious processing, orthographic activation in L2 spoken word recognition depends on proficiency in L2 (Veivo and Järvikivi, 2013). In the current study, we investigated how orthographic information affects L2 learners of different proficiency levels when it is overtly available. Furthermore, we were interested in the time-course of activation of this information. For these reasons, we used the visual world paradigm with printed words to track the matching process. The visual world paradigm has previously been used with bilingual subjects mostly with picture targets and to study parallel language activation and cross-linguistic competition (e.g., Spivey and Marian, 1999; Marian and Spivey, 2003a,b), the role of phonetic information and phonology (Ju and Luce, 2004; Weber and Cutler, 2004; Cutler et al., 2006; Marian et al., 2008) or the role of other factors like age of acquisition or language mode in this cross-linguistic competition (Canseco-Gonzalez et al., 2010). Some bilingual visual world studies have also considered the influence of proficiency on competition. Blumenfeld and Marian (2007) studied German-English bilinguals and concluded that high language proficiency and cognate status increased lexical competition. Chambers and Cooke (2009) contrasted proficiency and sentence context but observed no effect of proficiency on interlingual competition. More recently, the visual world paradigm has been used to study the role of control mechanisms (Mercier et al., 2014) and of language context (Mercier et al., 2016) in bilingual spoken word processing.

To our knowledge, the visual world paradigm with printed words has not been used previously to a great extent with bilinguals. Mishra and Singh (2014) studied cross-linguistic lexical competition with Hindi-English bilinguals. Their participants listened to L1 Hindi or L2 English sentences with embedded target words while they saw a visual display containing the phonological neighbor of the translation equivalent of the target word and three filler words. These cross-lingual phonological neighbors attracted significantly more looks than the filler items in both L1-L2 and L2-L1 directions. The authors conclude that this parallel activation shows that translation equivalents in the non-target language are automatically activated even when the two languages use different scripts. Their results show that orthographic information is activated during spoken word processing and, further, that this activation is language non-selective. In a subsequent study, the same authors showed that the effects were more pronounced and appeared earlier in high proficiency bilinguals than lower proficiency bilinguals (Mishra and Singh, 2016).

In the visual world task with printed words, the potential referents have to be read in the search for the referent that matches with the spoken target. The psycholinguistic grain size theory (PGST, Ziegler and Goswami, 2005, 2006) predicts that different degrees of consistency between phonology and orthography lead to relying on different grain sizes in the development of lexical representations. According to the PGST, these differences in grain size are also reflected as differences in developmental reading strategies: In transparent orthographies like Finnish, reading strategies are based on smaller phonological units and correspondences between graphemes and phonemes, whereas in deep orthographies like English, these strategies are based on multiple size units. This can be, for example, correspondences between larger phonological units and strings of letters as well as whole-word representations, but also grapheme-phoneme correspondences to some degree. It has been suggested that these reading mechanisms developed during L1 reading acquisition might be so deeply entrenched that they would be transferable also to the reading and recognition of L2 words (Akamatsu, 1998), and that they could therefore even explain differences in orthographic activation observed in L2 spoken word recognition (Dornbusch, 2012). In a task where phonological and orthographic word forms have to be matched, learners with a very transparent orthography in their L1 might therefore use orthography differently from native speakers who are used to a considerably less transparent orthography.

As we have shown, cross-linguistic competition with bilinguals has been studied to some extent. However, there are no previous studies that focus on the time course of activation of intralinguistic orthographic information in spoken word recognition with late L2 learners and no studies that investigate the role of L2 proficiency in this activation. The present study was designed to investigate these aspects in greater detail.

## Current study

In the current study, we tracked the activation of orthographic information in a visual world task where the participants matched spoken targets with printed referents in the presence of orthographically or phonologically similar competitor words. We investigated how the presence of phonological and orthographic overlap between the target and the competitor would influence the matching process and tracked the time course of this information. We were further interested in the role of L2 proficiency in this activation. To this end, we studied Finnish (L1) learners of French (L2) from a wide range of proficiency levels and contrasted them with an L1 French speaking control group.

We carried out two visual world eye-tracking experiments with printed words as referents. Participants' eye movements were recorded while they were instructed to click on the target word appearing on the computer screen with three other words (a competitor and two unrelated distractors). The visual display of four words was presented 200 ms before the target word onset. We used targets with two types of competitors differing in the amount of word initial phonological overlap (Experiment 1) and with two types of competitors differing in the amount of word initial orthographic overlap (Experiment 2). We focused on word initial overlap of the printed referents because word initial information is known to weigh more heavily in the spoken word recognition than word final information. For instance, Allopenna et al. (1998) showed with eye-tracking that competitors with a word initial overlap with the targets (e.g., target: beaker—competitor: beetle) compete for recognition more strongly and longer than competitors with rhyme overlap (e.g., target: beaker—competitor: speaker). Also, word initial phonetic mismatch has been shown to block lexical access unless the mismatch is very small (e.g., Marslen-Wilson et al., 1996; Frauenfelder et al., 2001). This is why we wanted to contrast the targets with competitors that always had a matching onset, i.e., shared both the first grapheme and the first phoneme with the targets. However, by grouping the competitors to two conditions with a different word initial overlap we hoped to shed light on the importance of orthographic information in mapping spoken forms into their written counterparts.

If mainly phonological information is used in the matching between spoken and written word forms (*phonological hypothesis*), we should observe less looks to the targets and a delayed mapping between spoken and written word forms in the presence of competitors with a longer word initial phonological overlap (e.g., <*base*> [ba:z] vs. <*bague*> [bag] or <*base*> [ba:z] vs. <*bain*> [b$\tilde{\varepsilon }$]). If mainly orthographic information is used to match spoken word forms to printed referents (*orthographic hypothesis*), we should observe less looks to the targets and a delayed mapping between spoken and written word forms in the presence of competitors with a longer word initial orthographic overlap (e.g., <*mince*> [m$\tilde{\varepsilon }$s] vs. <*mite*> [mit] or <*mince*> [m$\tilde{\varepsilon }$s] vs. <*mythe*> [mit]).

Unlike with L1 listeners, there is much more variation in the skills of L2 listeners at different proficiency levels. As explained above, they may have deficits in phonological knowledge at the sublexical level which show as problems in recognizing L2 sounds, and, consequently, as increased competition at the lexical level (see Weber and Cutler, 2004; Weber and Broersma, 2012), which can influence the matching of spoken and written word forms. Also, the process of learning the grapheme-to-phoneme conversion (GPC) rules may still be in progress. In the current task, phonological and orthographic word forms were both explicitly available. In this type of task, even beginning learners can be expected to perform successfully, but due to inaccurate phonological knowledge they might complete it slower than more advanced learners. This task type could also narrow down the difference between L1 listeners who might be more familiar with the spoken than the written word forms and L2 listeners who might be less familiar with the spoken forms but have been more exposed to the orthographic forms.

Many studies on orthographic activation in L2 spoken word processing focus on English. However, because English orthography is opaque and very inconsistent both from sound to spelling (feedback consistency) and from spelling to sound (feedforward consistency) perspectives, this may have consequences with respect to the theoretical conclusions drawn from these studies. Therefore, there is a need for studies exploring orthographic activation in languages with different orthography-phonology mappings. We studied L1 Finnish learners of L2 French and contrasted them with an L1 French control group. We chose Finnish because it has a very transparent and consistent orthography in both directions. French, in turn, has a much more opaque orthography which is relatively feedforward consistent but on the other hand highly feedback inconsistent. This means that even if it is quite difficult to deduce how unfamiliar spoken words are spelled in French, it is relatively easy to guess how even unknown printed words are pronounced on the basis of how different combinations of letters are pronounced.

We first set out to investigate the use and activation of phonological information in the matching of spoken and written word forms in Experiment 1.

### Experiment 1

#### Method

##### Participants

L2 participants: Sixty-four students from the University of Turku participated for course credit or volunteered to participate. This study was carried out in accordance with the recommendations of University of Turku ethics committee with written informed consent from all subjects. All subjects gave written informed consent in accordance with the Declaration of Helsinki. All participants had Finnish as their L1 and none of them had an early bilingual background. They reported no hearing impairment or language deficits and had normal or corrected-to-normal vision. The L2 participants represented a wide range of proficiency levels in French from beginners to near-natives (self-evaluations on the CEFR-scale ranging from A1 to C2). In addition to their L1, the participants knew between 2 and 7 other languages and had French as their L2–L7 in order of acquisition (L4 for 48% of the participants). Participant-related information for the L2 group is summarized in Table 1.

**Table 1:** **Participant background information for the L2 group in Experiments 1 and 2 (***n*** = 64)**.

**L2 Participant background variable**	**Min**	**Max**	**Mean**	**Median**
Age	19	49	23.3	22
Age of onset for French	5	45	15.6	14
L2 proficiency = self-evaluations on CEFR scale^*^	5	28	16.3	17
Length of residence in a French speaking country (weeks)	0	98	11.8	2
Exposure to French (h /week)	1	43	11.5	9
Order of acquisition for French	2	7	4.0	4
Number of foreign languages spoken	2	7	5.3	5

* *Scores on CEFR-scale: 1–5 = A1, 6–10 = A2, 11–15 = B1, 16–20 = B2, 20–25 = C1, 25–30 = C2*.

L1 participants: Twenty-four L1 speakers of French were paid to participate. They were either Erasmus exchange students at the University of Turku (10) or students at Aix-Marseille University in France (14). They reported no hearing impairment or language deficits and had normal or corrected-to-normal vision. None of the participants had an early bilingual background. Participant-related background information for the L1 control group is summarized in Table 2.

**Table 2:** **Participant background information for the L1 group in Experiments 1 and 2 (***n*** = 24)**.

**L1 Participant background**	**Min**	**Max**	**Mean**	**Median**
Age	18	51	25.4	21
Number of foreign languages spoken	1	4	3.3	3

##### Materials

The visual displays consisted of four words: target, competitor and two distractor words. In Experiment 1, there were 20 target words (e.g., <base>) that were associated with competitors having either a higher degree of word initial phonological overlap (e.g., <bague> [bag]) or a lower degree phonological overlap (e.g., <bain> [b$\tilde{\varepsilon }$]) with the targets. The nucleus vowel of the higher overlap competitor (<bague> [bag]) was always pronounced similarly to the target (<base> [ba:z]), whereas the nucleus vowel of the lower overlap competitor (<bain> [b$\tilde{\varepsilon }$]) was always pronounced differently from the target (<base> [ba:z]). Both type of competitors, therefore, had the same degree of word initial orthographic overlap but a different degree of phonological overlap with the target (+ORTH+PHON vs. +ORTH−PHON). Each target with its competitors was associated with two orthographically, phonologically and semantically unrelated distractors. As the targets, competitors and distractors could not be matched for written length within the selection criteria, we allowed one letter difference in length for each display. Higher and lower overlap competitors were matched as well as possible for written frequencies reported in Lexique 3 (New et al., 2001). The mean frequency was 87.5 per million for the higher overlap competitors and 87.9 per million for the lower overlap condition. The distractors were matched for frequency with the target. The mean frequency of the target words was 99.5 per million; the mean frequency of the distractor words was 88.5 per million. The 20 target words are listed in Supplementary Table 1. In addition to the targets, 50 filler sets were constructed. In 20 of the filler sets, there was a word initial orthographic and phonological overlap between the distractors: a lower degree phonological overlap between distractors in 10 filler sets and a higher degree phonological overlap between distractors in 10 filler sets. This was to prevent participants from developing test-taking strategies and from recognizing target word displays by the orthographic similarity between targets and competitors. The remaining 30 filler sets consisted of four words with no orthographic, phonological or semantic overlap.

Target words were recorded embedded in a fixed French instruction sentence to click on the target word (e.g., *cliquez sur le mot base*). The recordings were conducted digitally using the SANAKO Lab100 hardware in the Learning, Age, and Bilingualism laboratory (LAB-lab) of the University of Turku. A female native speaker of French, unaware of the objectives of the study, read the sentences in a randomized order with a brief prosodic break preceding each target word. The recorded sentences were edited using PRAAT (Boersma and Weenink, 2011). The mean duration of the target words was 611 ms.

##### Design and procedure

Experiment 1 consisted of 70 trials (20 target word displays, 20 manipulated filler displays and 30 filler displays). Each trial consisted of listening to the spoken instruction sentence and then choosing the correct word with a mouse click from the four word displays on the computer screen. The position of the targets and competitors in the display was randomized for each display. For the 20 target word displays, the two types of competitors were counterbalanced between the two experimental lists so that each list comprised an equal number of higher (10) and lower (10) phonological overlap competitors. The 70 trials were presented to each participant in a randomized order, and the participants were assigned to the two experimental lists in order of appearance within their proficiency level group.

**Figure 1:**
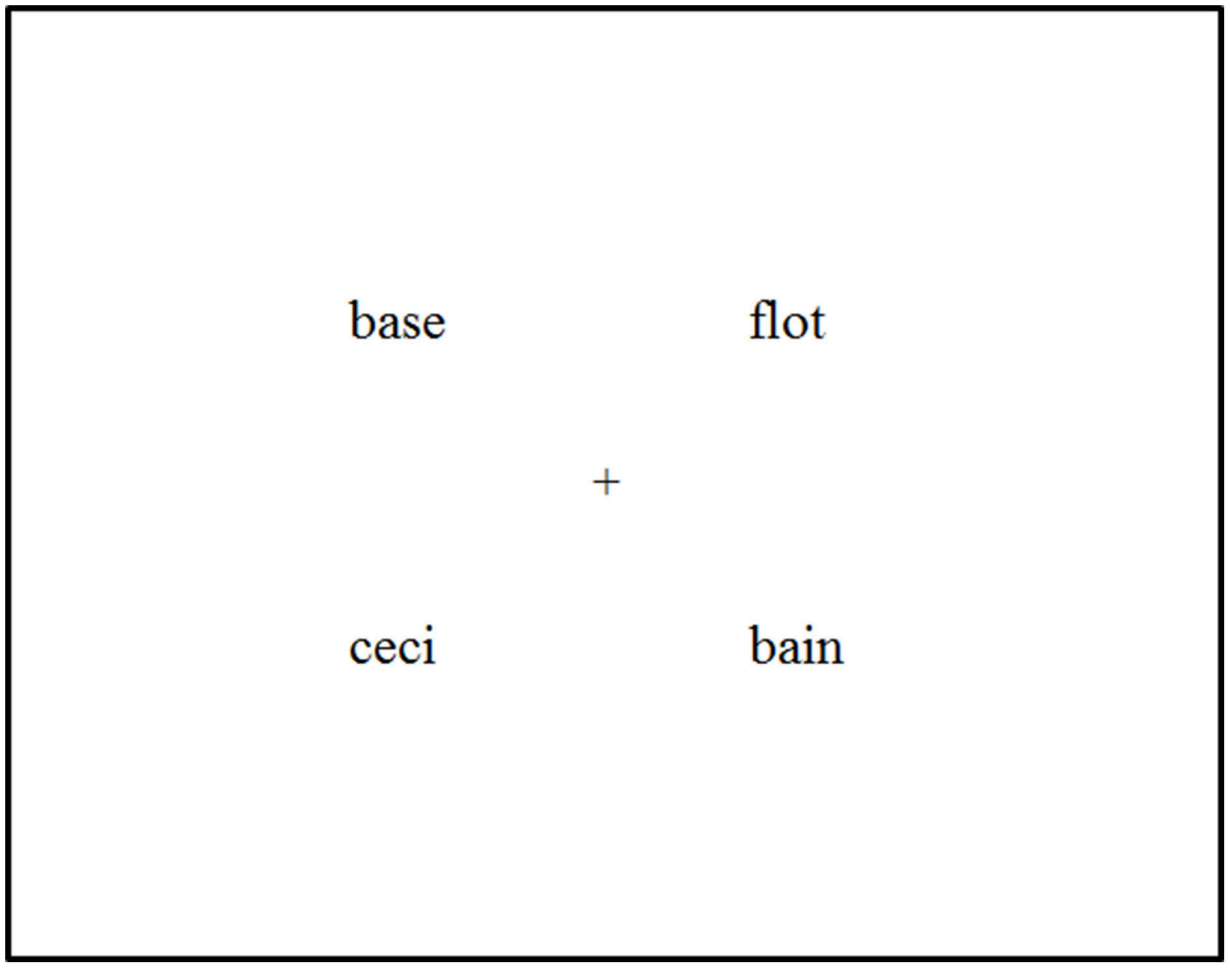
**Example of the visual display in the experiments**.

Participants' eye movements were monitored using a headmounted SR Research EyeLink II eye-tracking system (www.sr-research.com) with the participants from University of Turku and an SR EyeLink 1000 Tower Mount system with the 14 participants from the University of Aix-Marseille. Both systems were sampling at 500 Hz. Each experimental session started with the calibration of the eye tracker. In the beginning of each trial, a drift correction was performed with the presentation of a small cross in the center of the screen, and when the participant fixated on the cross, the experimenter accepted the gaze accuracy. Then the participant listened to a spoken instruction via the headphones to click on the target word. The printed words appeared on the screen 200 ms before the onset of the target word in the sentence, as in McQueen and Viebahn (2007), Huettig and McQueen (2007), and Salverda and Tanenhaus (2010). This timing ensured that the participants did not have time to read the words and access phonology via orthographic word forms before hearing the target word. The visual display was formally identical to the one used by Salverda and Tanenhaus (2010): the four words were written in lowercase Times New Roman font with each word being ~3–4° wide and its center appearing ~8° from the center of the screen (see Figure 1).

Before the actual experiment, participants were familiarized with the task by a practice block consisting of 10 displays with 4 unrelated words. After the practice block they conducted Experiments 1 and 2 in a counterbalanced order.

#### Results and discussion

Before the analyses, 30 erroneous responses (1.7%) were removed from the data. Of these erroneous clicks on the competitor, 5 trials were in the L1 group (1.0%) and 25 trials in the L2 learner group (2.0% of the data).

**Figure 2:**
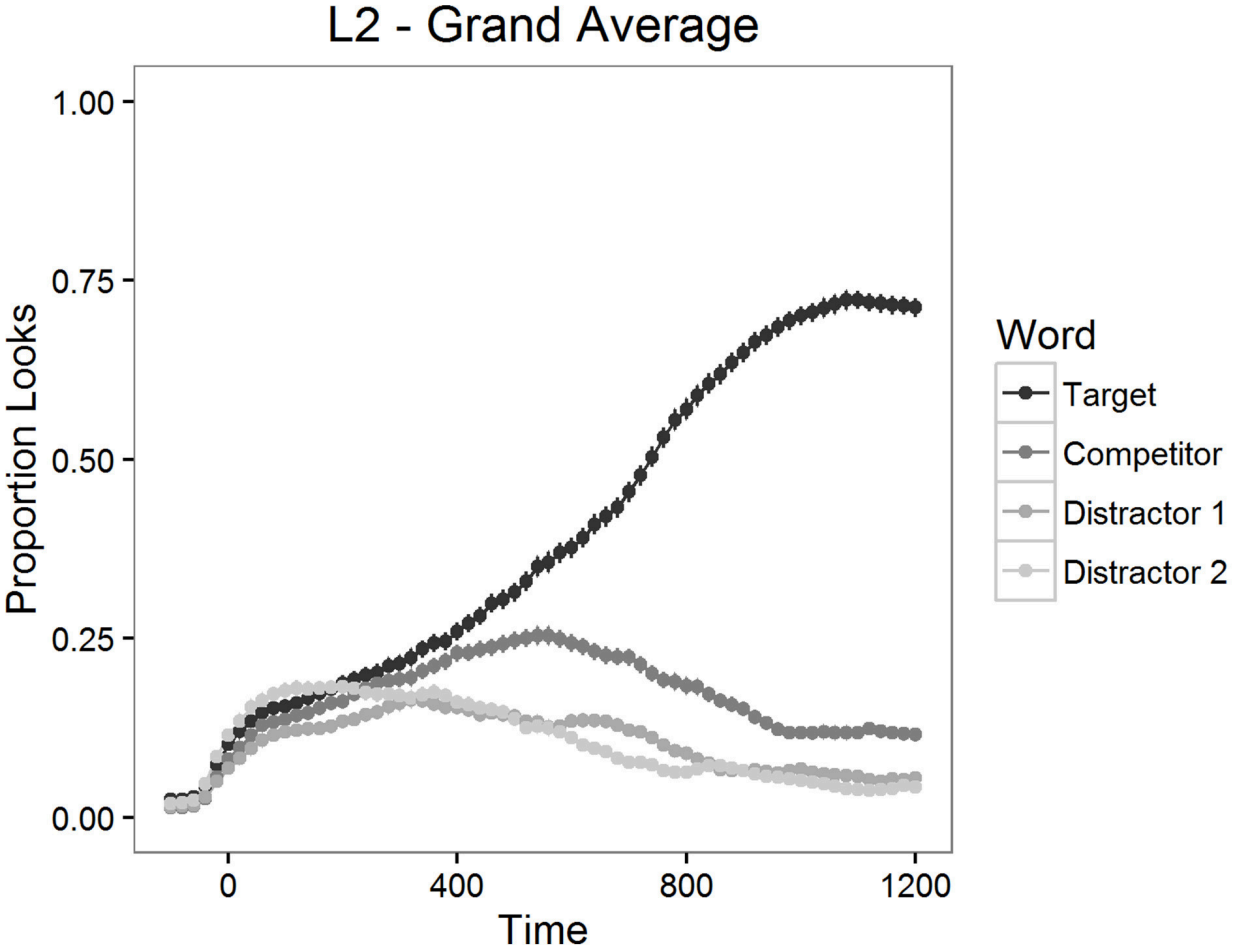
**Mean proportion of looks to each type of word in Experiment 1, L2 participants**.

**Figure 3:**
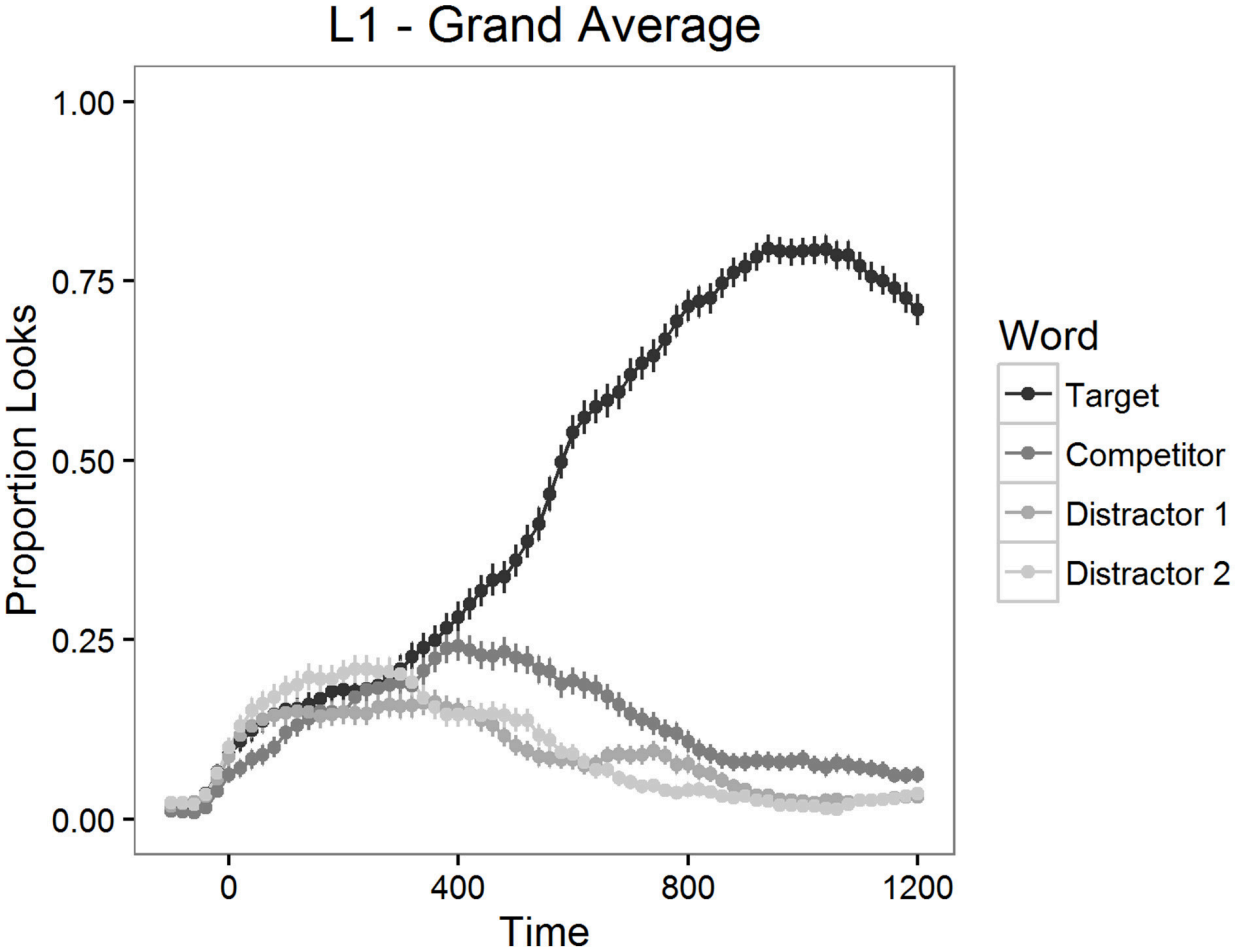
**Mean proportion of looks to each type of word in Experiment 1, L1 participants**.

The proportion of looks to the targets, to the competitors and to the distractors was calculated for each item and for each participant in 20 ms time bins. The mean proportions of looks for the 1200 ms time period starting from target word onset are depicted in Figure 2 for L2 participants and in Figure 3 for L1 participants.

As can be seen in Figures 2, 3, looks to the competitor and to the distractors started to diverge from target looks around 300 ms after the target word onset and reached the asymptotic level around 1000 ms post onset. We therefore examined competition effects within this 700 ms time window. Before statistical analyses, the proportions of looks in the 20 ms time bins were logit-transformed (see Barr, 2008). Logit-transformation distributes the values symmetrically around zero and provides an unbounded measure for the analysis. These values were then averaged within the window mentioned above.

First, we analyzed the differences between the looks to each type of word in the display (targets, competitors, and distractors) with paired *t*-tests. There were significantly more looks to targets than to competitors both in the L2 learner group [*t*_(1254)_ = 20.21, *p* < 0.001] and in the L1 group [*t*_(474)_ = 14.56, *p* < 0.001]. Also, participants fixated competitors significantly more often than distractors both in the L2 learner group [*t*_(1254)_ = 12.60, *p* < 0.001] and in the L1 group [*t*_(474)_ = 9.08, *p* < 0.001]. These results confirmed that competitors were inducing competition, as expected.

Next, we analyzed the looks to targets more in detail to test the mappings between spoken and written word forms. We started by analyzing all L2 and L1 participants together and fitted a linear mixed effects regression model (*lmer* in R) to the logit-transformed proportion of looks. We used a model structure with Participants and Items as a crossed-random factor, and Phonological Overlap condition (+ORTH+PHON vs. +ORTH−PHON), Group (L2 learners vs. L1 control group), Experimental List and Trial as fixed-effect predictors. The model was fitted with a backwards step-wise elimination procedure where the predictor variables that did not significantly improve the model as indicated by likelihood ratio testing were removed one by one. The inclusion of random slopes for participant by overlap and for items by group was justified by the likelihood tests (Bates et al., submitted). There was a significant main effect of Group [*estimate* = −0.77, *t*_(67.17)_ = −2.96, *p* = 0.004], which indicated that L1 participants found the targets significantly better than L2 participants in the analyzed time window. The effect of Phonological Overlap as such was not significant, but there was a significant interaction between Group and Phonological Overlap [*estimate* = 0.59, *t*_(86.72)_ = 2.40, *p* = 0.019]. Multiple comparisons of means indicated that there was a significant difference between groups only for targets when they were presented with higher phonological overlap competitors (target: <base> [ba:z] competitor: <bague> [bag]) for which the L1 participants were more likely to find the targets than L2 participants [*p* = 0.014].

Next, we analyzed the looks to the targets in the two groups of participants separately. We checked also for competitor activation but we did not find significant effects in competitor looks. Therefore, we focused on target fixations because it was the target looks, the relative ease of finding the target, that were affected by our experimental manipulation and our participant groups. Participants and Items were used as a crossed-random factor, and Phonological Overlap condition, Experimental List and Trial as fixed-effect predictors. For both groups, List and Trial were removed in the model fitting process. In the L1 group, the resulting model did not show a significant effect for Phonological Overlap [*estimate* = −0.32, *t*_(432.10)_ = −1.85, *p* = 0.065]. In the L2 group, the likelihood tests justified including random slopes for participants by overlap in the model. The degree of Phonological Overlap between targets and competitors proved to be significant [*estimate* = 0.26, *t*_(63.52)_ = 2.02, *p* = 0.048]. This main effect of Phonological Overlap indicated that if the nucleus vowel of the competitors was pronounced differently than in the targets (target: <base> [ba:z] vs. competitor: <bain> [b$\tilde{\varepsilon }$]), L2 learners found the targets significantly better in the analyzed time-window than if the nucleus vowel of the competitor was pronounced similarly to the targets (target: <base> [ba:z] vs. competitor: <bague> [bag])^1^. This suggests that a longer word initial phonological overlap inhibited the mapping process more than only a shared onset with the targets, and shows that in L2 spoken word recognition the search for printed referents is mediated by L2 phonology.

We next moved on to examine the time-course of the recognition process in L2 learner group more in detail. In order to analyze the possible influence of proficiency for L2 learners who represented a wide range of proficiency levels from A1 to C2, we used generalized additive mixed modeling (GAMM) (Hastie and Tibshirani, 1990; Wood, 2006). GAMM is well suited for the analysis of visual world time-course data, because it does not assume a linear relationship between predictors as does ANOVA or linear regression. Therefore, it is capable of handling non-linearities inherent in the visual world eye-tracking data (see Figures 2, 3). Additionally, given the time-series nature of the data, GAMM allows for the control of autocorrelation. Autocorrelation relates to the correlation between data points in a time-series; a measurement at time point t correlates to differing degrees with a measurement at time point t-i, depending on the lag. In this way, it is possible to model the possibly non-linear effect of time, which can then be allowed to interact with other continuous predictors (e.g., Proficiency) (see also Baayen et al., 2015, 2016).

To investigate the time-course of the word recognition process in the L2 learner group, we analyzed looks to targets within an unaveraged time window of 800 ms from 200 ms to 1000 ms. As in the *LMER* analyses, the response variable of the models was the logit-transformed proportion of looks to the target. The input variables (Event, List, Trial, Time ^*^ Proficiency, and Difference surface for high Phonological Overlap) were fit to the response variable (Looks to Targets) with by-Event random intercepts. Here, Event represents the unique combination of Participant and Item. By-Event random intercepts allow for the possibility that some combinations of Participants and Items may be more likely than others to attract target looks. A non-linear functional relationship between Time and Proficiency was allowed, using a tensor product (Wood, 2006). For the smoothing parameter of Time and Proficiency, a difference surface was included for Phonological Overlap, using a tensor product (Wood, 2006; Baayen, 2010). This difference surface represents the deviation values which get added to the surface for Time and Proficiency to result in the surface for Phonological Overlap. Additionally, an AR-1 correlation parameter, rho = 0.895, was estimated from the data and included to control for autocorrelation in the time series. The model was fit using a backwards step-wise elimination procedure with the inclusion of predictors in the model being evaluated using two criteria. The first criterion was the estimated *p*-value of the smoothing parameter or parametric component. The estimated *p*-value of the smoothing parameter or parametric component indicates whether or not the functional form of the predictor is different from zero. If greater than the conventional alpha level of 0.05, the predictor was considered for removal. The second criterion was Maximum Likelihood (ML) score comparison between variant models (Zuur et al., 2009). Upon removal of the predictor, the ML score of that model was compared to that of the model containing the predictor in question (i.e., the full model), indicating whether or not the inclusion of the predictor significantly improved model likelihood. Through the model fitting process, Trial and the Difference surface for high Phonological Overlap were removed. Chi-square tests comparing the ML scores of the model variants justified the inclusion of Proficiency [$\chi_{\left(3\right)}^{2}$ = 14.59, *p* < 0.001] in the model. Thus, the final model contained the following predictors: Event, Experimental List, Time ^*^ Proficiency, and explained 25.4% of the deviance. The statistics for the parametric and smooth terms of the model with best fit are presented in Table 3.

**Table 3:** **GAMM with best fit for L2 participants' target looks in Experiment 2: parametric coefficients and estimated degrees of freedom (Edf), reference degrees of freedom (Ref. ***df***), ***F***-values and ***p***-values for the tensor products**.

**Parametric coefficients**	**Estimate**	**Std. Error**	***t*-value**	***p*-value**
(Intercept)	−0.591	0.073	−8.140	<0.001
**Smooth terms**	**Edf**	**Ref**. ***df***	***F*****-value**	***p*****-value**
Smooth: Items and Subjects	371.83	1128.00	0.493	<2e-16
Tensor: Time, Proficiency	13.82	17.20	54.626	<2e-16
Tensor: Time, Proficiency ^*^ Overlap condition	11.90	14.44	2.242	= 0.004

**Figure 4:**
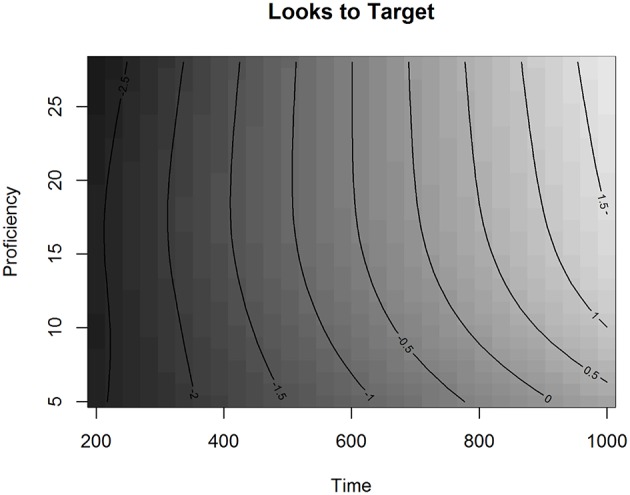
**The effect of Proficiency over Time in Experiment 1 in the L2 group, fitted values**.

The effect of Proficiency on the Looks to Targets over Time is visualized in Figure 4.

In Figure 4, the effect of Proficiency over Time is presented as a regression surface where the contour lines represent the estimated Looks to Targets fitted by the model. Shades of darker gray depict less looks to the target, whereas shades of lighter gray depict more looks to the target. By looking at the gradual change of color in the figure we can see that listeners were more likely to be looking at the target as time progressed. The plot also shows that as proficiency decreases, the contour slopes become less steep. This indicates that lower proficiency learners were slower in finding the targets than higher proficiency learners. We can also see that proficiency affected the ease of finding the targets particularly in the lower end of the proficiency scale, especially for proficiency scores under 15 (CEFR-levels A1, A2, and B1). For instance, if we compare the change over time in target looks for proficiency scores 10 and 20, we can see that looks to the target increase faster for participants with the higher score. In the GAMM analysis, the effect of overlap was not significant, neither was the interaction between proficiency and overlap. This shows that the general phonological effect observed in the *LMER* analysis of the L2 group was not modulated by proficiency nor did it significantly change over the analyzed time-window. This result suggests that, for Finnish learners of French, the visual search for printed referents is mediated via phonology at all proficiency levels.

In Experiment 1, we contrasted targets and two types of competitors with identical word initial orthographic but different phonological overlap (+ORTH+PHON vs. +ORTH−PHON). Both L1 and L2 participants looked significantly more to competitors than to unrelated distractors. This finding suggests that orthographically similar word forms compete more strongly for recognition than word forms without an orthographic overlap. In the L2 group, we also observed a significant effect for the difference in the phonological overlap. If the nucleus vowel of the competitor was pronounced similarly to the nucleus vowel of the target, the competitor impacted the mapping of the target more negatively than competitors where the nucleus vowel was pronounced differently from the targets. We did not observe this same effect of phonological overlap in the L1 group. This suggests that L1 French listeners and L1 Finnish listeners were not using the same type of information in the matching process. Proficiency in the L2 did not influence the effect of overlap over time. It was significant, however, for the speed of the matching process: the higher the proficiency in the L2, the faster the targets were found. Also, L1 participants found the targets significantly faster than L2 participants.

We next moved on to investigate the role of orthographic information in the matching process. We wanted to evaluate how the degree of word initial orthographic overlap between competitors and targets would affect the matching process when the amount of phonological overlap was held constant. As in Experiment 1, we were interested in the role of proficiency in the L2 in the use of this information.

### Experiment 2

#### Method

##### Participants

Participants were the same as in Experiment 1. For this reason, the order of presentation for the two experiments was counterbalanced between the participants.

##### Materials

Similar to Experiment 1, the visual displays comprised four words: target, competitor and two distractor words. Experiment 2 consisted of 18 target words. Each target word (e.g., <mince>) was associated with either a higher degree orthographic overlap competitor (e.g., <mite>) or a lower degree orthographic competitor (e.g., <mythe>). The nucleus vowel of the target word was always pronounced differently than either of the competitors (<mince> [m$\tilde{\varepsilon }$s] vs. <mite> [mit] / <mythe> [mit]), which were always homophonous. Thus, each type of competitor had the same phonological mismatch in the nucleus vowel but a different amount of orthographic overlap with the target (+ORTH−PHON vs. −ORTH−PHON). Each target and the two competitor types were associated with two orthographically, phonologically and semantically unrelated distractor words. It was not possible to match targets, competitors and distractors perfectly for written length, so as in Experiment 1, we allowed a one letter difference between the words in each display. High and low overlap competitors were matched for written word frequency reported in Lexique 3 (New et al., 2001). For the high overlap competitors the mean frequency was 71.0 per million and for the low overlap competitors 66.8 per million. Distractors (56.5/million) were matched for frequency with the targets (57.5/million). The 18 target word displays are listed in Supplementary Table 2. In addition to the target word displays, 46 filler displays were constructed. In 18 of these filler displays, there was an orthographic overlap between the two distractors. As with the target word displays, in the filler displays, 9 contained low overlap between the distractors, and 9 contained high overlap between distractors. This manipulation was done because, as in Experiment 1, we wanted to prevent the participants from recognizing the target sets on the basis of similarity between two words in the display. In the remaining 28 filler displays there was no orthographic, phonological or semantic overlap between the four words. The procedure of recording of the targets was identical to that in Experiment 1. Mean acoustic duration of the target words was 564 ms.

##### Design and procedure

The design and procedure were identical to those in Experiment 1 apart from the number of trials which was 64 (18 target word displays, 18 manipulated filler displays and 28 filler displays).

#### Results and discussion

**Figure 5:**
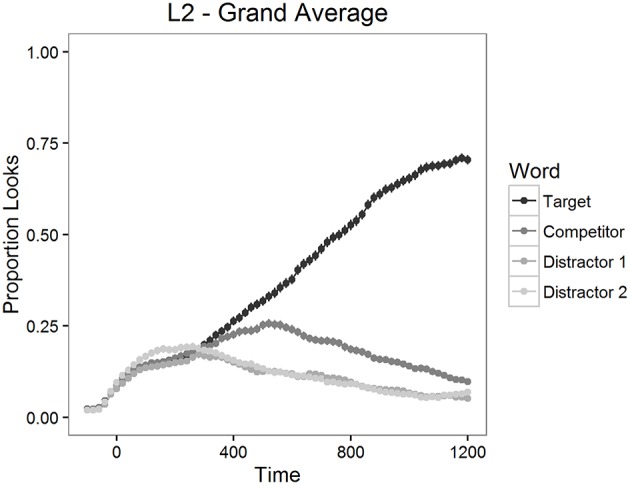
**Mean proportion of looks to each type of word in Experiment 2, L2 participants**.

**Figure 6:**
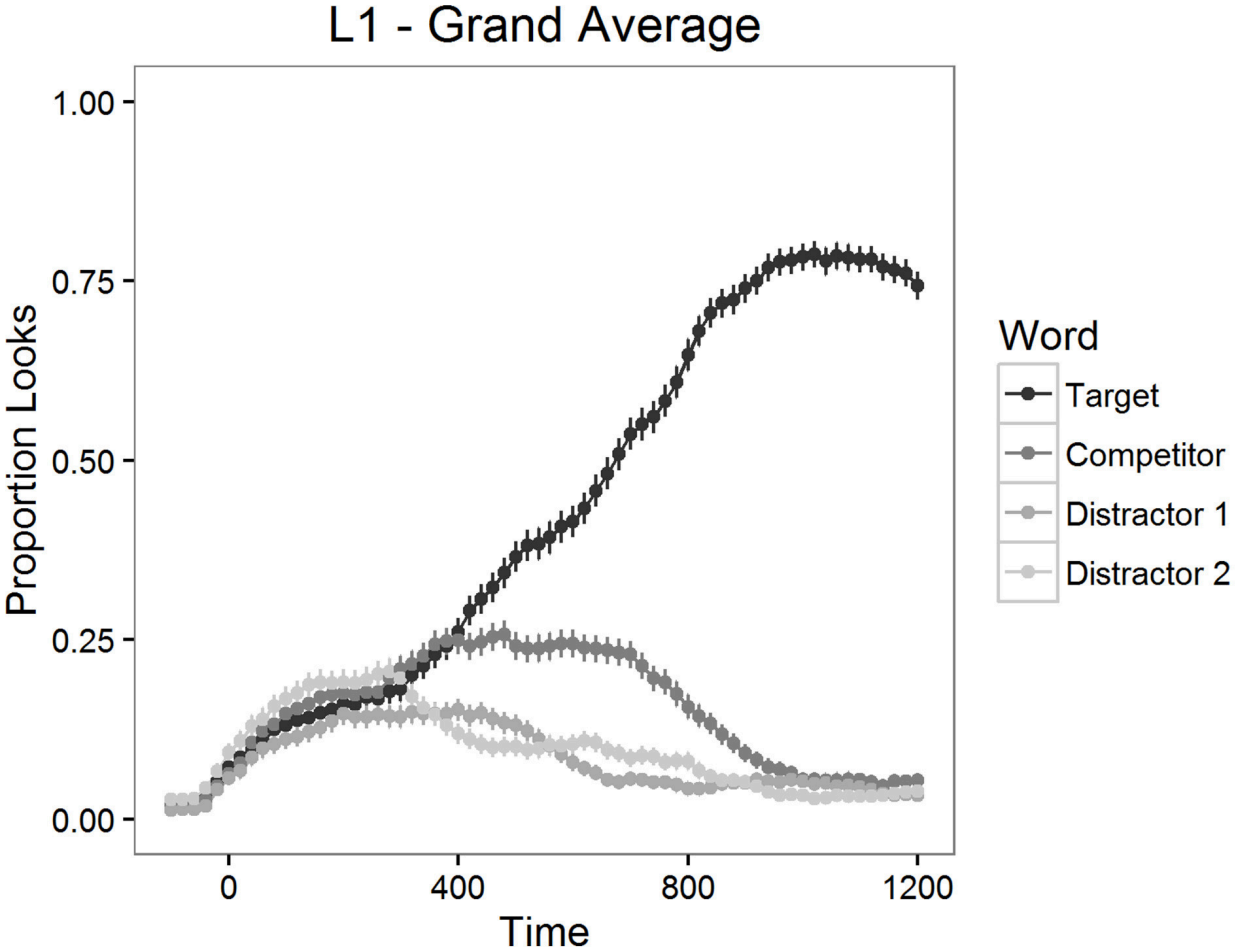
**Mean proportion of looks to each type of word in Experiment 2, L1 participants**.

First, erroneous responses were removed from the data, representing 1.3% of the whole dataset (21 trials): 1.7% of the L2 group data (20 trials) and 0.2% of the L1 group data (1 trial). The number of looks to each type of word in the display was calculated in 20 ms time bins for each trial and for each participant to determine the mean proportion of looks to each word. The mean proportion of looks to the target, to the competitor and to the distractors for a 1200 ms period starting from the target word onset is depicted in Figure 5 for L2 group and in Figure 6 for L1 group.

Proportions of looks to distractors started to diverge from target and competitor looks at about 300 ms after the onset of the target word (Figures 5, 6). Therefore, we analyzed competition effects within a 700 ms time window starting 300 ms after target word onset by contrasting the averaged looks to each type of word. As in Experiment 1, the proportions of looks in the 20 ms time bins were logit-transformed before statistical analyses to give an unbounded measure. These values were then averaged within the window mentioned above.

As in Experiment 1, we started by analyzing the differences between the looks to targets, competitors and distractors for all the participants with paired *t*-tests. The differences between looks to targets and competitors as well as between looks to competitors and distractors were significant in both groups. In the L2 group, there were significantly more looks to targets than to competitors [*t*_(1131)_ = 21.20, *p* < 0.001] and to competitors than to distractors [*t*_(1131)_ = 12.17, *p* < 0.001]. Also, in the L1 group, targets attracted significantly more looks than competitors [*t*_(430)_ = 18.04, *p* < 0.001], and competitors attracted significantly more looks than distractors [*t*_(430)_ = 6.89, *p* < 0.001]. These results confirmed that competitors induced competition, as expected.

As the competition effects were showing in the target looks, we then continued to analyze the Looks to Targets for all participants with linear mixed-effects regression (*LMER*). We used Participants and Items as a crossed-random factor (Baayen et al., 2008), and Orthographic Overlap (+ORTH−PHON vs. −ORTH−PHON), Group (L2 learners vs. L1 control group), Experimental List and Trial as fixed-effect predictors. The model fitting procedure was the same as for Experiment 1. The likelihood-ratio test showed that adding random slopes for Item by Group improved the model fit significantly. The variables eliminated in this process were List, Trial and Orthographic Overlap. The model with the best fit showed a significant effect of Group [*estimate* = 0.67, *t*_(47.59)_ = −3.06, *p* = 0.004]. It indicated that L2 participants found the targets significantly less well than L1 participants (reference level) in the analyzed time window. No significant main effect was found for Orthographic Overlap, nor was there a significant interaction between the type of overlap and group that would suggest that the effect of overlap would be different in the two groups^2^.

We next moved on to analyze the looks to targets in the two groups of participants separately. We proceeded as above and fitted *LMER*-models to Looks to Targets in both groups. Participants and Items were used as a crossed-random factor, and Orthographic Overlap, Experimental List and Trial as fixed-effect predictors. The model fitting procedure was the same as above. List and Trial were removed in this fitting process for both groups. As in the combined analysis, Orthographic Overlap between targets and competitors was not significant in either the L2 group [*estimate* = 0.06, *t*_(1052.00)_ = 0.59, *p* = 0.55] or the L1 control group [*estimate* = −0.08, *t*_(389.90)_ = −0.44, *p* = 0.66].

These results indicated that competitors with a longer word initial orthographic overlap did not impact the mapping of the target more than competitors with shorter orthographic overlap in either group, when there was a phonological mismatch between the target and the competitor after the onset. In other words, these results suggest that the degree of orthographic overlap was not as decisive for lexical competition to impact processing as was phonological overlap between targets and competitors. In a task such as the present where auditory words had to be matched with their orthographic forms, the candidates that shared both the orthographic and phonological onset with the targets were activated. However, the search for a potential referent seems to have been driven more by phonology than by orthography, as the phonetic mismatch in the nucleus (Experiment 1) affected it more than the length of orthographic overlap.

**Figure 7:**
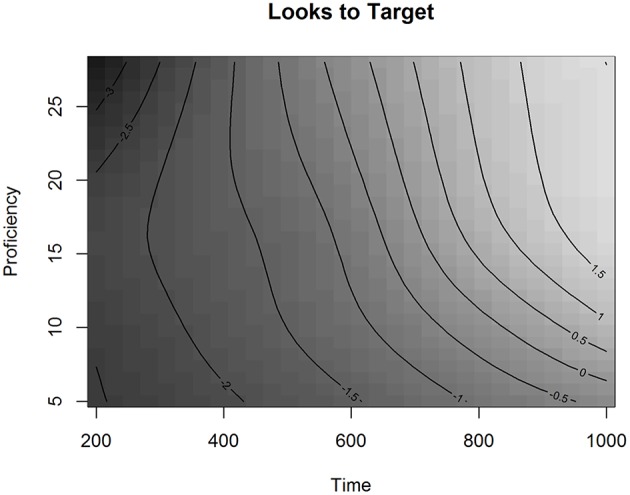
**The effect of Proficiency over Time in Experiment 2 in the L2 group, fitted values**.

Finally, as in Experiment 1, we analyzed the time-course of spoken word recognition and the possible effect of variability in L2 proficiency in the L2 learner group in more detail. We analyzed Looks to Targets within a 800 ms analysis window starting 200 ms after the target word onset. The model structure was as in the GAMM analysis of Experiment 1 with Event, Experimental List, Trial, Time ^*^ Proficiency, and Difference surface for high Orthographic Overlap as input variables. Event represents the unique combination of Participant and Item, as in Experiment 1. Input variables were fitted to the response variables with by-Event random intercepts. A non-linear functional relationship between Time and Proficiency was allowed, along with a difference surface for high Orthographic Overlap. The difference surface represents the deviation values from the surface for Time and Proficiency which result in the surface for high Orthographic Overlap. Lastly, an AR-1 correlation parameter, rho = 0.895, was estimated and included to control for autocorrelation in the time-series. The model fitting procedure followed that of Experiment 1. Through this process, List and Trial were removed. Chi-square tests comparing the ML scores of the model variants justified the inclusion of Proficiency [$\chi_{\left(6\right)}^{2}=47.19$, *p* < 0.001] and Orthographic Overlap [$\chi_{\left(6\right)}^{2}$ = 8.65, *p* = 0.008] as input variables in the model. Thus, the final model contained the following predictors: Event, Time * Proficiency, and Difference surface for high Orthographic Overlap, and explained 26.1% of the deviance. The statistics for the parametric and smooth terms of the model with the best fit are presented in Table 4. The significant interaction between Time and Proficiency is represented in Figure 7.

**Table 4:** **GAMM with best fit for L2 participants' target looks in Experiment 1: parametric coefficients and estimated degrees of freedom (Edf), reference degrees of freedom (Ref. ***df***), ***F***-values and ***p***-values for the tensor products**.

**Parametric coefficients**	**Estimate**	**Std. Error**	***t*-value**	***p*-value**
(Intercept)	−0.591	0.073	−8.140	<0.001
**Smooth terms**	**Edf**	**Ref**. ***df***	***F*****-value**	***p*****-value**
Smooth: Items and Subjects	371.83	1128.00	0.493	<2e-16
Tensor: Time, Proficiency	13.82	17.20	54.626	<2e-16
Tensor: Time, Proficiency * Overlap condition	11.90	14.44	2.242	= 0.004

The interaction between Time and Proficiency is presented in Figure 7 as a regression surface. The contour lines in the plot represent the estimated looks to targets fitted by the model. Lighter shades of gray represent greater looks to target, darker shades of gray represent lesser looks to target. The plot shows that with lower proficiency participants the contour slopes are shallow whereas with higher proficiency participants the slopes are steeper. This shows again that higher proficiency learners found the targets faster than lower proficiency learners. For example, if we compare the time-course of target looks for proficiency scores 10 and 20 between 400 ms and 600 ms, we can see that lower proficiency participants are slower in finding the targets. Additionally, by observing the shape of the slopes we can see that the upper half of the participants with a proficiency score above 15 seems to be more homogeneous for speed than the lower half of the participants. This effect indicates that higher proficiency learners are processing the auditory input more efficiently, resolving the target sooner in time. In the lower half, the effect of proficiency is more gradient, in other words, decreased proficiency slows the mapping in a gradient fashion.

**Figure 8:**
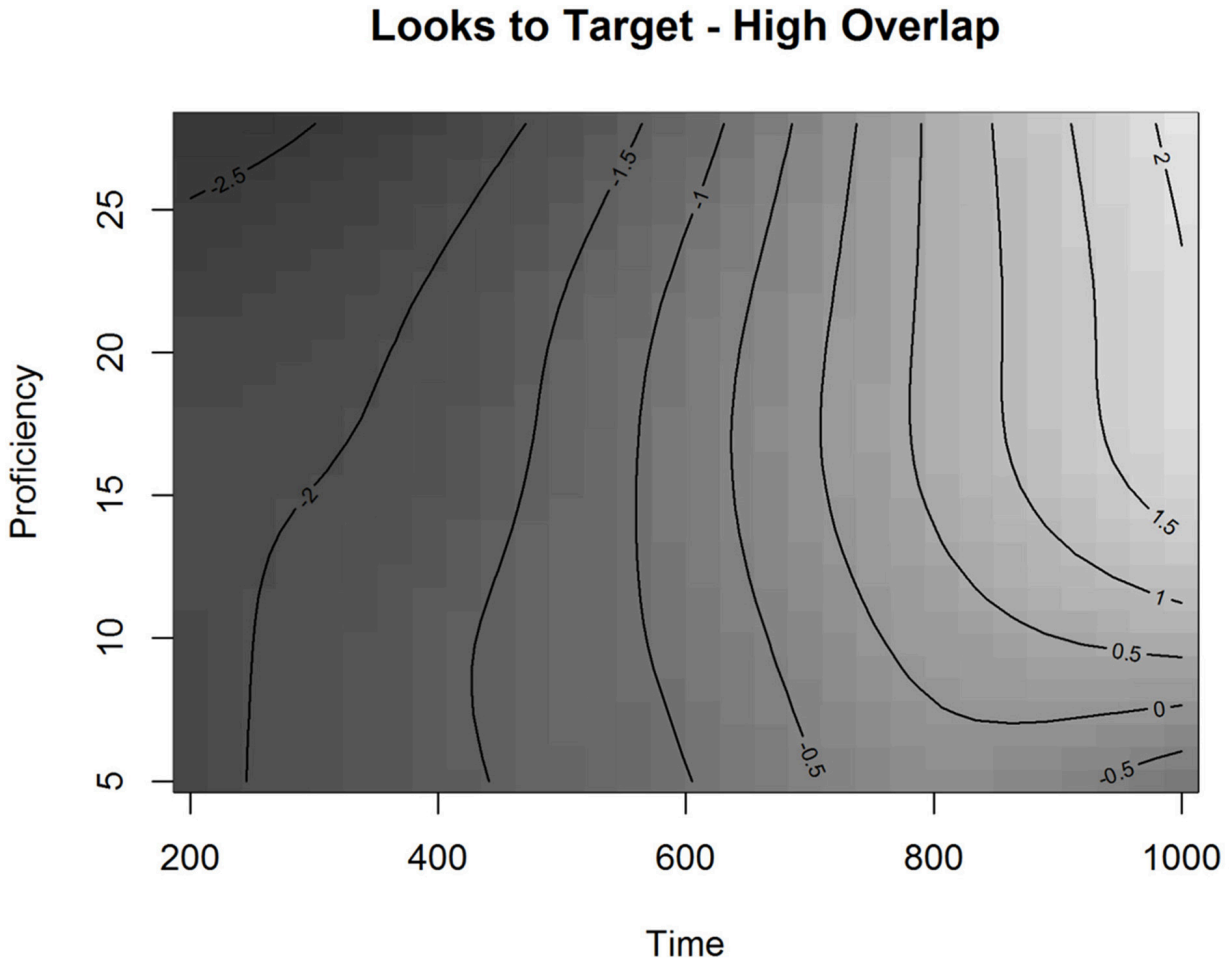
**Partial effect of High orthographic Overlap as a function of Time and Proficiency in Experiment 2 in the L2 group**.

**Figure 9:**
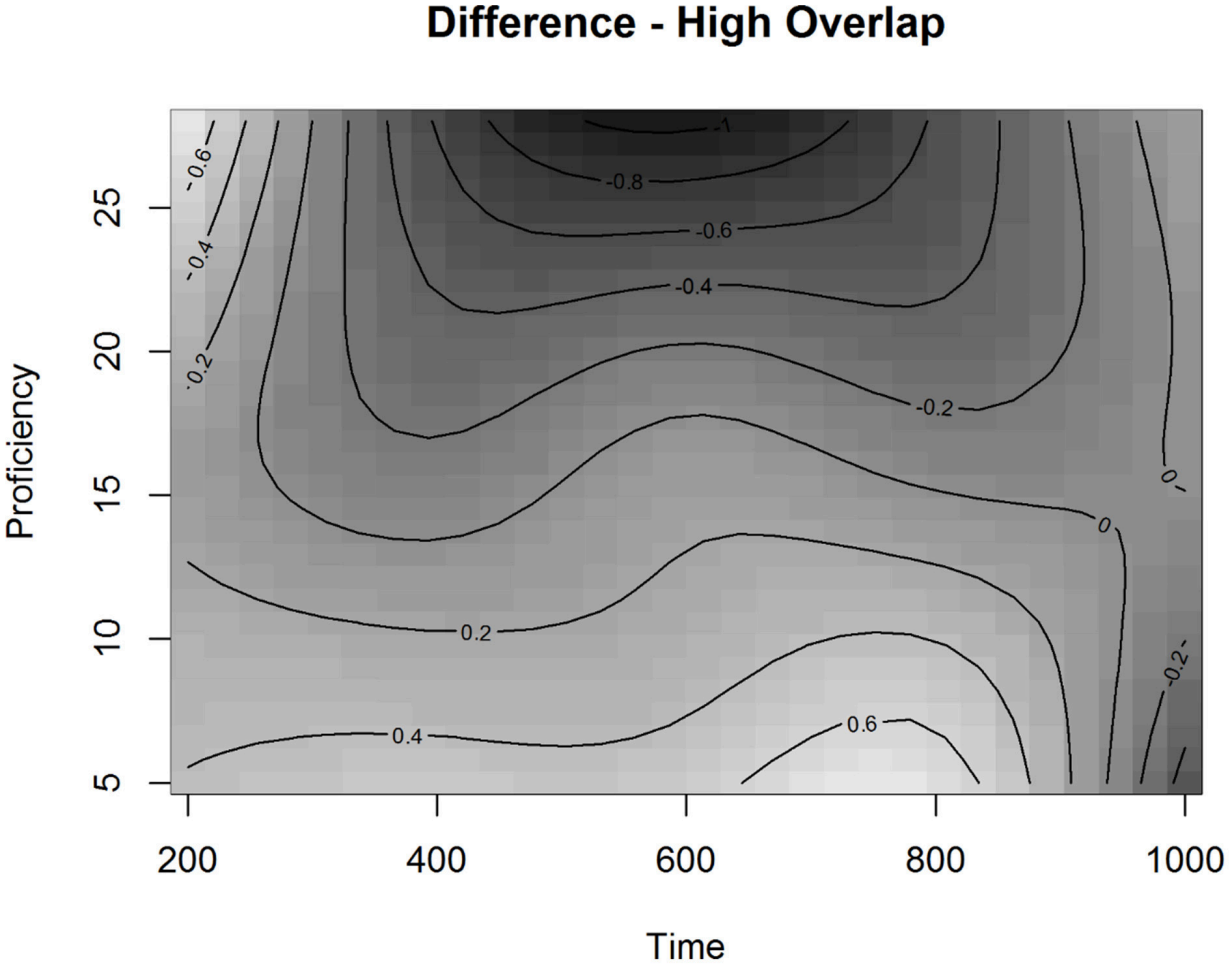
**Partial effect of the Difference surface for high Orthographic Overlap as a function of Time and Proficiency in Experiment 2 in the L2 group**.

Interestingly, we also observed a significant adjustment for High Overlap for the smoothing parameter of Time and Proficiency. Figure 8 presents the fitted surface for High Overlap items as a function of Time and Proficiency. The contour lines represent the estimated Looks to Targets in the presence of competitors with a longer orthographic overlap fitted by the model. The estimated effect in Figure 8 indicates that when the competitor has a higher orthographic overlap with the target, learners across the proficiency scale behave more similarly early in time with the effect of proficiency emerging at ~700 ms after the onset. After this time point, higher proficiency learners are more likely to be on the target than lower proficiency learners. This shows that while higher proficiency learners demonstrate a stronger influence of high overlap, they are still able to resolve it. To examine this effect in more detail, we plotted the partial effect of the Difference surface for high Orthographic Overlap as a function of Time and Proficiency (Figure 9). This surface is added to the surface in Figure 7 to produce the surface in Figure 8.

As can be seen in Figure 9, looks to the target for higher proficiency learners are reduced in the range of 400 to 700 ms in the presence of orthographic overlap. This suggests that a higher orthographic overlap is inhibiting the matching process for these learners in this time range. This, in combination with the plot presenting the fitted values (Figure 8), indicates that the late effect of proficiency relates to participants with proficiency values above 15. This result is compatible with our results of the GAMM analysis in Experiment 1, which also showed that there is a cutoff point between B1- and B2-levels of the CEFR-scale with participants of the lower half behaving in the task differently from the higher half.

These results suggest that, with L1 Finnish learners of L2 French, proficiency level in the L2 significantly influences the way orthographic information is used in spoken word recognition. Higher proficiency learners found the targets better than lower proficiency participants. However, when the orthographic overlap was high, the better the L2 proficiency the fewer looks to targets there were in the time-window from 400 to 700 ms after the target word onset. This means that competitors with a higher orthographic overlap disrupted the mapping process more for higher proficiency learners than for lower proficiency learners in this time-window. This suggests that in the matching between spoken forms and printed referents higher proficiency learners are using orthographic information differently from lower proficiency learners.

Experiment 2 contrasted competitors with a different degree of orthographic overlap but an identical phonological mismatch between targets and competitors (+ORTH−PHON vs. −ORTH−PHON). Both the L2 learner group and the L1 control group produced significantly more looks to the competitors than to the unrelated distractors. This indicates that similarity in the word form, a shared onset (same grapheme and same phoneme), influenced the mapping in relation to the unrelated distractors. However, we did not find a significant main effect for orthographic overlap: a longer word initial orthographic overlap did not affect looks to target more than a mere onset overlap when both groups of participants were analyzed together or separately. However, when the time-course of the recognition process was analyzed for the L2 group, we observed an interesting effect of proficiency for the Overlap over Time. Higher proficiency learners found the targets less well in a time-window from 400 to 700 ms if the competitors had a longer word initial orthographic overlap with the targets. In contrast, lower proficiency learners found the targets better with higher than lower orthographic overlap competitors. This suggests that orthographic information plays a different role in the recognition process depending on the proficiency level in L2. The level of proficiency in the target language was also significant in the recognition speed. L1 participants found the targets significantly faster than L2 participants, and within the L2 group, more proficient L2 learners found the targets faster than less proficient learners.

We now move on to discuss the results of both experiments more in detail.

## Discussion

In the present study, we contrasted targets and competitors varying in length of word initial phonological overlap (Experiment 1) and in length of word initial orthographic overlap (Experiment 2). The difference between the two overlap conditions was not significant in the L1 control group in either of the experiments. However, we observed a general phonological effect in L1 Finnish learners of L2 French: target words were fixated significantly less in the presence of competitors with a longer word initial phonological overlap (<base> vs. <bague>) than with a shorter phonological overlap (<base> vs. <bain>). The degree of orthographic overlap was not significant in the L2 group as a whole: longer word initial orthographic overlap did not have a more significant impact on the mapping process than a mere onset overlap in the analyzed time-window. Yet, when the time-course of activation of the orthographic information in the L2 group was analyzed more in detail, we discovered that the effect of orthographic overlap over time depended on proficiency in the L2. Higher proficiency learners found the targets less well in a time-window from 400 to 700 ms after target onset if the competitors had a longer word initial orthographic overlap with the targets (<mince> vs. <mite>) than if the orthographic overlap was shorter (<mince> vs. <mythe). This increased effect of orthography was observed only for learners in the upper half of the proficiency scale, whereas for lower proficiency learners competition was decreased.

These results indicate that when printed words are presented briefly before the spoken words in the visual world paradigm, L1 Finnish participants at all proficiency levels use phonological information to match the printed referent with the spoken French word. These results are in line with the phonological hypothesis and suggest that with Finnish late learners of L2 French, phonology has a role to play in the visual search for printed referents. Our results are, in this respect, different from the results of Salverda and Tanenhaus (2010), who did not find any significant difference for the degree of phonological overlap in L1 English listeners with the same printed word paradigm. Instead, they observed a rapid orthographic competition effect and conclude that for L1 English listeners, visual search for printed referents is mediated via orthographic information activated immediately upon hearing spoken words. In contrast, the current study did not find this type of orthographic effects in the L1 French control group either^3^.

As predicted, the likelihood and speed of finding the targets depended on proficiency. As a group, L1 participants found the targets better than L2 participants in both experiments within the analyzed time-window. Additionally, within the L2 group, more proficient L2 learners found the targets faster than less proficient learners. Moreover, in both experiments the speed of finding the targets depended significantly more on proficiency in the lower half of the proficiency scale—the lower the score, the longer it took to find the targets—whereas participants in the upper half of the proficiency scale were more homogeneous in this respect. The cutoff point for this proficiency effect was in the middle of the CEFR-scale between the B1 and B2 levels. That proficiency affects the speed of finding the targets only in the lower half of the proficiency scale suggests that the gradual process of acquiring the skills and information needed in the matching task is still ongoing up to this cutoff point, above which processing is more homogenous and does not depend on level of proficiency. This effect was particularly salient in Experiment 1 where we observed a general phonological effect for all proficiency levels. The task used in the present study does not tease apart whether this effect reflected the acquisition process of L2 sounds or the acquisition of grapheme-phoneme correspondences in L2. While it likely results from acquiring knowledge of both, further research is needed to make more detailed conclusions about this matter.

We observed a general phonological effect in the matching of spoken and written word forms with L1 Finnish learners of L2 French who have a very consistent and transparent orthography in their L1. According to the Psychological Grain Size Theory, different orthographies lead to different reading strategies which can also be transferable to the L2 (Akamatsu, 1998; Ziegler and Goswami, 2005). The differences in reading strategies might partly explain why L1 English listeners show orthographic effects in the matching task (see Salverda and Tanenhaus, 2010), but L1 French listeners do not. Readers of a feedforward inconsistent orthography like English might be using larger units and base the matching more on visual information even at the whole word level. Readers of a much more feedforward consistent orthography like French are used to activating the phonology more and therefore are not influenced more by competitors with a larger orthographic overlap than competitors with smaller orthographic overlap if the two mismatch phonologically with the targets. There is evidence that the writing system of the L1 does not affect processing of written words in the L2 (Lemhöfer et al., 2008) and that L2 readers develop processing mechanisms that are based on the L2 orthographic system (de Groot et al., 2002). However, our findings are in line with the view that the processing mechanism of written words in L2 reflects that of L1. Especially lower proficiency learners who were slower in the matching process might be relying on similar reading strategies as beginning L1 readers of a transparent orthography. The matching process is slowed down if participants are trying to decode L2 orthography based on one-to-one relations between graphemes and phonemes, like in their L1. This may lead to greater activation of phonology also in the L2 in a task where orthographic and phonological word forms have to be matched.

Furthermore, L2 learners show a great variability in vocabulary size and lexical knowledge, and for unfamiliar word forms, L2 participants may be basing the matching only on sublexical correspondences. As stated above, this is unlikely to prevent success in the task, but it may affect the degree of activation of orthographic information during the task performance. Ventura et al. (2004) suggest that processing at the lexical level is necessary for orthographic effects to rise in spoken language processing, as they did not observe orthographic consistency effects for pseudowords. The same result was confirmed by Pattamadilok et al. (2014). Unfamiliar L2 word forms can in this respect be paralleled to pseudowords because neither of them have lexical representations. Further research contrasting words and pseudowords in a similar matching task could shed light on how the level of processing affects orthographic activation.

Even though we did not observe a general orthographic effect in Experiment 2 in the L2 group as a whole, our results indicated that the effect of orthographic overlap depended on proficiency. The participants in the upper half of the proficiency scale looked less to the target words in the presence of higher orthographic overlap competitors, while the participants in the lower half looked to the target words more. The effect of orthographic overlap for higher proficiency learners was observed between 400 and 700 ms. These same learners showed a significant phonological effect in Experiment 1, so they based the matching also on phonological information. At this time point, however, also orthographic information is relevant for them. This co-activation of both forms of representation could be explained by a qualitative difference in lexical knowledge reflecting a better interaction between phonological and orthographic knowledge in more proficient learners. For these advanced learners, even phonologically mismatching competitors can induce more competition in the recognition process if they have a longer orthographic overlap with the competitors (<mince>– <mite>) than competitors with a shorter orthographic overlap (<mince>– <mythe>).

As this orthographic effect was not found with L1 French listeners, we suggest that this effect may result from the process of co-structuration of orthographic and phonological knowledge in the formal instruction. Unlike L1 listeners, L2 listeners are exposed to written language early on in the acquisition process. This leads to a strong orthographic component in the learners' lexical knowledge. However, because of the deficits in the phonological knowledge mentioned above, there is probably also less interaction between the two representation modes until a certain level of proficiency is reached. Our results indicate that below that level, a higher orthographic similarity does not affect the matching process, whereas when this level is reached, the matching of spoken and printed word forms can be mediated via orthography even when targets and competitors are phonologically mismatching. This finding suggests that learners in the upper half of the proficiency scale have integrated orthographic and phonological knowledge better than learners in the lower half of the scale. The cutoff point for the orthographic effect was the same as the cutoff point for the effect of proficiency on processing speed in Experiment 1. This indicates that the acquisition of L2 spoken word recognition is not linear and that spoken word processing may be based on a different balance between orthography and phonology in higher and lower proficiency learners. This cutoff point may also reflect different mechanisms of orthographic activation at different proficiency levels. When learners have reached a certain proficiency level, they may start to process L2 words based on abstract representations containing both orthographic and phonological information instead of relying on sublexical correspondences.

To conclude, the results of the present study indicate that L2 learners with a formal instruction background use the available orthographic information differently depending on their proficiency in the L2. The study shows that when orthographic information is overtly available, higher proficiency L2 learners can base the visual search for potential referents on orthography in addition to phonological information. An interesting direction for further research would be to investigate the role of L2 proficiency for orthographic activation in spoken word recognition with L2 listeners in tasks where orthography is not explicitly present or is masked.

## Author contributions

OV: The design of the experiments, analysis, interpretation of data, drafting and revising the text, final approval of the text and agreement to be accountable for all aspects of the work in ensuring that questions related to the accuracy or integrity of any part of the work are appropriately investigated and resolved. JJ: The design of the experiments, analysis, interpretation of data, drafting and revising the text, final approval of the text and agreement to be accountable for all aspects of the work in ensuring that questions related to the accuracy or integrity of any part of the work are appropriately investigated and resolved. VP: Analysis and interpretation of data, drafting and revising the text, final approval of the text and agreement to be accountable for all aspects of the work in ensuring that questions related to the accuracy or integrity of any part of the work are appropriately investigated and resolved. JH: Interpretation of data, revising the text, final approval of the text and agreement to be accountable for all aspects of the work in ensuring that questions related to the accuracy or integrity of any part of the work are appropriately investigated and resolved.

## Funding

The testing of the L1 participants in Aix-Marseille University was made possible by a travel grant to the first author from the Emil Aaltonen Foundation.

### Conflict of interest statement

The authors declare that the research was conducted in the absence of any commercial or financial relationships that could be construed as a potential conflict of interest.

## Footnotes

^1^As parafoveal processing at fixation would favor word-initial information when presented to the right of fixation, we tested the effect of left/right screen presentation, but the side of the screen the competitor was on (right or left) was not a significant predictor [*estimate* = 0.395, *t*_(16.103)_ = 1.562, *p* = 0.138]. We thank the reviewers for suggesting this possibility.

^2^As in Experiment 1, we tested the possible effect of left/right screen presentation, but the side of the screen the competitor was on (right or left) was not a significant predictor [*estimate* = 0.138, *t*_(17.984)_ = 0.495, *p* = 0.626]. We thank the reviewers for suggesting this possibility.

^3^It is also possible that as the number of subjects in the L1 control group was smaller than in the L2 group, there was not enough experimental power for the effects to become significant, but given the results, we think this is unlikely. We thank the reviewers for suggesting this possibility.
