# Supplementary material for: Orthographic Activation in L2 Spoken Word Recognition Depends on Proficiency: Evidence from Eye-Tracking
Source: Front Psychol. 2016 Jul 27;7:1120. doi: 10.3389/fpsyg.2016.01120 (PMC4961715; doi:10.3389/fpsyg.2016.01120)
Supplement: Supplementary file 1 [file Table1.DOCX]

Table 1: Targets and competitors in Exp. 1.

| **target** | **frequency** | **higher overlap competitor** | **frequency** | **lower overlap competitor** | **frequency** |
| --- | --- | --- | --- | --- | --- |
| base | 44.46 | bague | 22.4 | bain | 83.04 |
| belge | 8.99 | berge | 16.5 | beau | 620.1 |
| botte | 44.93 | bordé | 1.28 | boule | 61.22 |
| fente | 15.61 | femme | 996 | ferme | 58.04 |
| guêpe | 6.42 | guère | 111 | guise | 20.61 |
| large | 107.5 | laque | 2.7 | laide | 26.82 |
| maison | 575.3 | maigre | 62.7 | maillot | 18.99 |
| malin | 20.2 | manie | 18.4 | maire | 13.85 |
| miette | 17.16 | mienne | 41.3 | mieux | 398.4 |
| plaire | 139.8 | plaie | 24.3 | plaint | 67.84 |
| porte | 617.4 | pomme | 82.4 | pouce | 35.34 |
| rame | 11.55 | race | 34.9 | rang | 58.24 |
| sabre | 17.03 | salon | 101 | saute | 4.66 |
| serre | 6.62 | seize | 31.4 | seuil | 49.86 |
| soie | 51.62 | soif | 35.5 | soin | 68.24 |
| tempe | 29.46 | tendu | 37.6 | texte | 43.24 |
| tiers | 14.93 | tiède | 44.7 | tiens | 81.89 |
| tome | 7.09 | tort | 55 | toux | 12.23 |
| venger | 24.46 | vente | 19 | venin | 3.11 |
| verre | 230.1 | vexer | 11.8 | veuf | 32.7 |
| **mean** | 99.5 |  | 87.5 |  | 87.9 |

Supplementary Table 2: Targets and competitors in Exp. 2.

| **target** | **frequency** | **higher overlap competitor** | **frequency** | **lower overlap competitor** | **frequency** |
| --- | --- | --- | --- | --- | --- |
| denier | 1.62 | dense | 11.7 | danse | 35.27 |
| fier | 58.18 | fin | 315 | faim | 75.95 |
| vaincu | 8.51 | vaine | 57 | veine | 35.41 |
| rendu | 14.73 | renne | 1.15 | reine | 33.78 |
| pesé | 70.88 | peau | 188 | pot | 48.04 |
| char | 27.57 | chair | 102 | cher | 133.7 |
| sage | 31.15 | saut | 17 | seau | 24.05 |
| tenue | 31.89 | tente | 26.2 | tante | 118.4 |
| linge | 47.3 | lisser | 11.4 | lycée | 38.78 |
| terme | 59.73 | teint | 24.3 | thym | 2.09 |
| taille | 76.49 | taire | 140 | terre | 452.9 |
| mince | 78.51 | mite | 2.23 | mythe | 10.41 |
| gare | 84.53 | gai | 41.8 | guet | 7.7 |
| poids | 89.05 | pose | 19.9 | pause | 11.89 |
| toit | 91.76 | tôt | 127 | taux | 2.64 |
| sale | 102 | sain | 18.7 | sein | 84.05 |
| paix | 103.7 | pain | 105 | peint | 20.74 |
| vert | 145.1 | veau | 17 | vos | 180.3 |
| **mean** | 57.5 |  | 71.0 |  | 66.8 |
