# Supplementary material for: Orthographic Activation in L2 Spoken Word Recognition Depends on Proficiency: Evidence from Eye-Tracking
Source: Front Psychol. 2016 Jul 27;7:1120. doi: 10.3389/fpsyg.2016.01120 (PMC4961715; doi:10.3389/fpsyg.2016.01120)
Supplement: Supplementary file 2 [file Table2.DOCX]

Table 2: Targets and competitors in Exp. 2.

| **target** | **frequency** | **higher overlap competitor** | **frequency** | **lower overlap competitor** | **frequency** |
| --- | --- | --- | --- | --- | --- |
| denier | 1.62 | dense | 11.7 | danse | 35.27 |
| fier | 58.18 | fin | 315 | faim | 75.95 |
| vaincu | 8.51 | vaine | 57 | veine | 35.41 |
| rendu | 14.73 | renne | 1.15 | reine | 33.78 |
| pesé | 70.88 | peau | 188 | pot | 48.04 |
| char | 27.57 | chair | 102 | cher | 133.7 |
| sage | 31.15 | saut | 17 | seau | 24.05 |
| tenue | 31.89 | tente | 26.2 | tante | 118.4 |
| linge | 47.3 | lisser | 11.4 | lycée | 38.78 |
| terme | 59.73 | teint | 24.3 | thym | 2.09 |
| taille | 76.49 | taire | 140 | terre | 452.9 |
| mince | 78.51 | mite | 2.23 | mythe | 10.41 |
| gare | 84.53 | gai | 41.8 | guet | 7.7 |
| poids | 89.05 | pose | 19.9 | pause | 11.89 |
| toit | 91.76 | tôt | 127 | taux | 2.64 |
| sale | 102 | sain | 18.7 | sein | 84.05 |
| paix | 103.7 | pain | 105 | peint | 20.74 |
| vert | 145.1 | veau | 17 | vos | 180.3 |
| **mean** | 57.5 |  | 71.0 |  | 66.8 |
